# Validity of age estimation methods and reproducibility of bone/dental maturity indices for chronological age estimation: a systematic review and meta-analysis of validation studies

**DOI:** 10.1038/s41598-022-19944-5

**Published:** 2022-09-16

**Authors:** V. Marconi, M. Iommi, C. Monachesi, A. Faragalli, E. Skrami, R. Gesuita, L. Ferrante, F. Carle

**Affiliations:** 1 Postgraduate School of Medical Statistics and Biometry, Department of Biomedical Sciences and Public Health, Università Politecnica delle Marche, 60126 Ancona, Italy; 2 Center of Epidemiology, Biostatistics and Medical Information Technology, Università Politecnica delle Marche, 60126 Ancona, Italy; 3 Department of Biomedical Sciences and Public Health, Università Politecnica delle Marche, 60126 Ancona, Italy; 4 Department of Pediatrics, Università Politecnica delle Marche, 60123 Ancona, Italy; 5 National Centre for Healthcare Research and Pharmacoepidemiology, 20126 Milan, Italy

**Keywords:** Medical research, Statistics

## Abstract

Several approaches have been developed to estimate age, an important aspect of forensics and orthodontics, using different measures and radiological examinations. Here, through meta-analysis, we determined the validity of age estimation methods and reproducibility of bone/dental maturity indices used for age estimation. The PubMed and Google Scholar databases were searched to December 31, 2021 for human cross-sectional studies meeting pre-defined PICOS criteria that simultaneously assessed the reproducibility and validity. Meta-estimates of validity (mean error: estimated age-chronological age) and intra- and inter-observer reproducibility (Cohen’s kappa, intraclass correlation coefficient) and their predictive intervals (PI) were calculated using mixed-effect models when heterogeneity was high (I^2^ > 50%). The literature search identified 433 studies, and 23 met the inclusion criteria. The mean error meta-estimate (mixed effects model) was 0.08 years (95% CI − 0.12; 0.29) in males and 0.09 (95% CI − 0.12; 0.30) in females. The PI of each method spanned zero; of nine reported estimation methods, Cameriere’s had the smallest (− 0.82; 0.47) and Haavikko’s the largest (− 7.24; 4.57) PI. The reproducibility meta-estimate (fixed effects model) was 0.98 (95% CI 0.97; 1.00) for intra- and 0.99 (95% CI 0.98; 1.00) for inter-observer agreement. All methods were valid but with different levels of precision. The intra- and inter-observer reproducibility was high and homogeneous across studies.

## Introduction

Age estimation, an important aspect of forensics and orthodontics, is often used when chronological age cannot be determined^1^. Indeed, estimating dental age in children is useful in several situations such as orthodontic treatment planning, forensic dentistry, and other clinical scenarios^2,3^. In living individuals, age estimation is a crucial and increasing forensic practice method due to widespread increases in individuals without identification documents and whose real age must clarified for criminal, civil, asylum, or old-age pension proceedings^4–9^. Age estimation is increasingly requested by judicial authorities to determine if the adult penal law should be applied according to legally-relevant age ranges^10^. Age estimation has also been used in professional sports, where age falsification could provide athletes with significant competitive advantages^11^.

Different methods are used to determine age using different measures and radiological examinations^10^, with the teeth and the hand-wrist commonly assessed. Teeth are one of the strongest structures in the human body and, together with the skeletal system, pass through a series of developmental changes that represent valid indices for age determination^12–16^. Skeletal maturity is based on radiography of specific structures such as the medial clavicular epiphysis cartilage^17–19^, pubic symphysis^20^, and the left hand-wrist area^10^. However, methods based on skeletal maturity are more variable and susceptible to error than methods based on tooth maturation^21–23^. Dental methods identify the stages of tooth mineralization in radiographs and code them according to predetermined scores^24^ or continuous measures^13,25^. The most common method for age estimation was published in 1973 by Demirjian, Goldstein, and Tanner^24^ and was subsequently modified by other authors. Demirjian’s method is based on eight developmental stages ranging from crown and root formation to apex closure of the seven left permanent mandibular teeth. A score is assigned at each stage and then the sum of the scores represents the subject’s dental maturity score (DMS). From this seminal paper, the DMS was used in regression equations to estimate the age of a subject.

Over the years, several different methods have been developed to increase the accuracy of age estimation. Technological developments in radiology have allowed more specific measurements to be made, increasing the accuracy of dental/skeletal maturation indicators^26–29^. There has also been a focus on refining age estimation methods to better predict chronological age^13,25,30^.

To consider a method "valid", it is necessary to proceed with its validation. Validation refers to the process of applying the age estimation method to a sample other than the one used to calibrate the method^31^. The sample can be external or a test set obtained by splitting the study sample into training and test sets. To evaluate the method’s validity, the distribution of errors between chronological and estimated age are then evaluated on this external sample or test set.

Inter-observer reliability is defined as the agreement between two or more observers, while intra-observer reliability is defined as the agreement of the same evaluator at two or more different time points. Cohen’s K statistic is commonly used for reliability assessments of categorical scales, while the intraclass correlation coefficient (ICC) or the concordance correlation coefficient (CCC) statistics are appropriate for continuous scales^32^.

Reference studies on forensic age estimation should report sex and ethnicity, two well-known factors associated with individual dental/skeletal maturity^33–35^, in addition to chronological age, bone age, the difference between bone age and chronological age, and intra-observer and inter-observer reproducibility^36^. While several literature reviews and meta-analyses have compared different age estimation methods^3,37–41^, to our knowledge there has yet to be a first meta-analysis also comparing validation and reproducibility. We aimed to assess the validity of age estimation methods based on bone or dental maturity indices and the reproducibility of these maturity indices, through meta-analysis of validation and reproducibility studies. Therefore, the Review questions are “What is the level of validity of age estimation methods based on bone and dental maturity indices? What is the level of reproducibility of bone and dental maturity indices?”

## Results

### Study selection

The literature search returned 51 articles from PubMed and 382 from Google Scholar (total 433). After removing duplicates (28 articles), the titles and abstracts were separately screened by two authors (VM and CM) to leave 75 eligible articles. After reading the full text, 59 articles were excluded because 31 articles did not validate the age estimation method; 10 articles focused only on assessing a threshold for 14- or 18-year-old subjects; and 18 articles had incomplete or unusable data.

**Figure 1:**
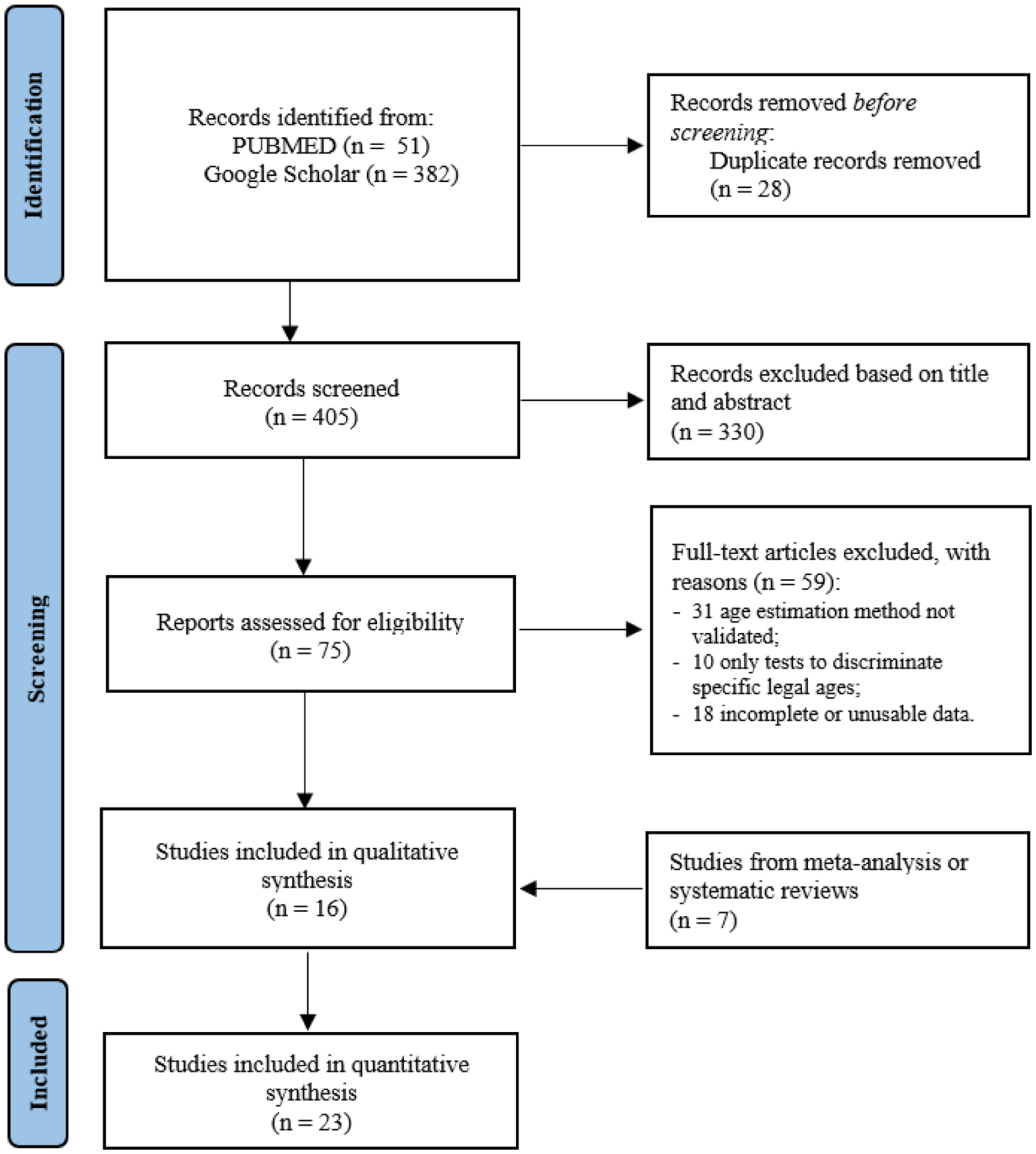
PRISMA flow diagram of the search results from the databases.

Sixteen studies were therefore included in the qualitative synthesis, and seven studies, which complied with the inclusion criteria, were also included after further examination of previous meta-analyses or systematic reviews to provide a total of 23 articles (Fig. 1).

### Characteristics of included articles

The characteristics of the 23 selected studies are detailed in Table 1. All studies adopted a cross-sectional design and were conducted in both university and hospital settings in 15 countries (Bosnia-Herzegovina^42,43^, Brazil^44^, China^45–47^, Colombia^48^, Egypt^49^, India^50–52^, Iran^53^, Italy^54^, South Korea^55^, Macedonia^56^, Malaysia^57,58^, Saudi Arabia^59^, Spain^60,61^, Sri Lanka^62^, and Turkey^63,64^). Sample sizes ranged from 70^52^ to 2641^61^subjects who underwent orthopantomography (21 articles^42–51,53,55–63^), or wrist and hand X-rays (2 articles^52,54^). The age range was from 1^52^ to 24 years^57^, and most studies (17 out of 23^42–44,46,48–51,53–56,58–60,63,64^) enrolled subjects aged 16 years or younger.

**Table 1:** The studies included in the meta-analysis. All studies reported the type of examination as “orthopantomography” except two^52,54^.

Author (year)^reference^	Study site	Sample size (male/female)	Age range (years)	Age estimation method	Total mean error (SD)	Male mean error (SD)	Female mean error (SD)	Inter-examiner agreement estimate (variance^§^)	Intra-examiner agreement estimate (variance^§^)
Ambarkova et al. (2013)^56^	Macedonia	N = 966 (481/485)	6.05–13.96	Demirjian	1.07 (0.96)	1.02 (1.02)	1.12 (0.9)	ICC = 0.89 (0.010)	ICC = 0.97 (0.001)
				Willems	0.42 (0.86)	0.52 (0.87)	0.33 (0.83)	ICC = 0.94 (0.003)	ICC = 0.97 (0.001)
Baghdadi et al. (2012)^59^	Saudi Arabia	N = 176 (91/85)	4–14	Demirjian		0.31 (0.93)	0.56 (0.81)	Inappropriate estimation method	
El-Bakary et al. (2010)^49^	Egypt	N = 286 (134/152)	5–16	Willems	0.15 (0.62)	0.29 (0.48)	0.14 (0.74)	Inappropriate estimation method	
				Cameriere	− 0.29 (1.04)	− 0.49 (1.03)	− 0.26 (1.21)		
Franco et al. (2013)^44^	Brazil	N = 462 (205/257)	5–16	Willems		0.24 (0.97)	0.04 (0.97)	Cohen’s k = 0.93	Cohen’s k = 0.9
Galić et al. (2010)^42^	Bosnia-Herzegovina	N = 1106 (509/597)	5–14	Demirjian		1.46 (1.26)	1.27 (1.27)		Cohen’s k = 0.82
Galić et al. (2011)^43^	Bosnia-Herzegovina	N = 1089 (498/591)	6–13	Cameriere		− 0.02 (0.71)	0.10 (0.71)	Cohen’s k = 1	Cohen’s k = 0.97
				Haavikko		− 0.09 (0.79)	− 0.23 (0.73)	Cohen’s k = 0.85	Cohen’s k = 0.98
				Willems		0.42 (0.77)	0.25 (0.89)	Cohen’s k = 0.81	Cohen’s k = 0.97
Javadinejad et al. (2015)^53^	Iran	N = 537 (264/273)	3.9–14.5	Demirjian	0.87 (1.00)	0.90 (1.01)	0.85 (0.98)		
				Willems	0.36 (0.87)	0.43 (0.82)	0.31 (0.91)		Cohen’s k = 0.96
				Cameriere	− 0.19 (0.86)	− 0.27 (0.85)	− 0.11 (0.87)		
				Smith	0.06 (0.63)	0.12 (0.83)	0.00 (0.81)		
Jayaraman et al. (2012)^45^	China	N = 266 (133/133)	2–21	Demirjian		− 0.25 (1.43)	− 0.23 (1.37)		Cohen’s k = 0.88
Kırzıoğlu & Ceyhan (2012)^64^	Turkey	N = 425 (212/213)	7–13	Nolla	− 0.54 (0.93)	− 0.53 (0.95)	− 0.57 (0.91)	ICC = 0.98 (0.008)	ICC = 0.95 (0.002)
				Haavikko	− 0.58 (0.80)	− 0.60 (0.80)	− 0.56 (0.81)		
				Demirjian	0.64 (0.89)	0.52 (0.86)	0.75 (0.90)		
Kumaresan et al. (2014)^58^	Malaysia	N = 426 (179/247)	5–16	Demirjian	− 0.97 (1.19)	− 0.98 (1.29)	− 0.97 (1.12)	Inappropriate estimation method	Inappropriate estimation method
				Willems	− 0.54 (1.28)	− 0.55 (1.40)	− 0.53 (1.20)		
				Nolla	− 0.54 (1.31)	− 0.50 (1.31)	− 0.57 (1.31)		
				Haavikko	1.31 (1.1)	0.94 (1.03)	1.59 (1.08)		
				Cameriere	0.41 (1.08)	0.44 (1.14)	0.39 (1.03)		
Lee et al. (2011)^55^	Korea	N = 1483 (754/729)	5–16	Demirjian	0.30 (0.81)	0.29 (0.75)	0.31 (0.87)	Inappropriate estimation method	Inappropriate estimation method
				Willems	− 0.17 (0.65)	− 0.15 (0.58)	− 0.19 (0.72)		
				Chaillet	− 0.35 (0.68)	− 0.38 (0.61)	− 0.31 (0.75)		
Melo & Ata-Ali (2017)^61^	Spain	N = 2641 (1322/1319)	7–21	Demirjian		0.99 (0.39)	0.72 (0.56)	ICC = 1 (0)	ICC = 1 (0)
				Nolla		− 0.27 (0.50)	− 0.16 (0.23)	ICC = 1 (0)	ICC = 1 (0)
Mohammed et al. (2015)^50^	India	N = 660 (330/330)		Demirjian	0.10 (1.63)	− 0.23 (1.87)	0.43 (1.27)	ICC = 0.9 (0.008)	ICC = 0.8 (0.026)
			6–16	Haavikko	− 2.90 (1.41)	− 2.84 (1.60)	− 2.96 (1.18)	ICC = 0.9 (0.008)	ICC = 0.8 (0.026)
				Nolla	0.47 (0.83)	0.32 (0.91)	0.62 (0.71)	ICC = 0.9 (0.008)	ICC = 0.8 (0.026)
				Willems	− 0.40 (1.53)	− 0.69 (1.69)	− 0.11 (1.30)	ICC = 0.9 (0.008)	ICC = 0.8 (0.026)
Nur et al. (2012)^63^	Turkey	N = 673 (342/331)	5–16	Demirjian	0.86 (1.26)	0.84 (1.36)	0.89 (1.15)	Inappropriate estimation method	Inappropriate estimation method
				Nolla	− 0.54 (1.4)	− 0.50 (1.38)	− 0.57 (1.43)		
Paz Cortés et al. (2020)^60^	Spain	N = 604 (302/302)	4–13	Willems	0.26 (0.91)	0.17 (0.88)	0.35 (0.93)		
				Demirjian	0.70 (0.95)	0.73 (0.94)	0.68 (0.95)	Cohen’s k = 0.98	Cohen’s k = 0.99
				Nolla	− 0.63 (0.97)	− 0.82 (0.98)	− 0.44 (0.93)	Cohen’s k = 0.98	Cohen’s k = 0.99
Ranasinghe et al. (2019)^62^	Sri Lanka	N = 668 (333/335)	8–17	Demirjian	0.19 (0.87)	0.18 (0.81)	0.21 (0.93)	Cohen’s k = 0.83	Cohen’s k = 0.92
				Willems	− 0.38 (0.84)	− 0.38 (0.85)	− 0.38 (0.84)		
				Blenkin & Evans	− 0.55 (1.04)	− 0.53 (1.02)	− 0.56 (1.05)		
Rivera et al. (2017)^48^	Colombia	N = 457 (240/217)	6–14	Cameriere		0.08 (0.68)	− 0.25 (0.65)	ICC = 0.96 (0.001)	ICC = 0.99 (0.001)
Santoro et al. (2012) ^54^	Italy	N = 535 (243/292)	7–15	Greulich-Pyle*		− 0.1 (1.3)	0.4 (1.0)	Inappropriate estimation method	Inappropriate estimation method
				Demirjian		1 (1.5)	1.1 (1.6)		
Singh et al. (2020)^51^	India	N = 900 (458/442)	10–16	Nolla	− 0.15 (0.46)	− 0.21 (0.53)	− 0.09 (0.35)	Fleiss’ k = 0.78	Fleiss’ k = 0.84
Tiwari et al. (2020)^52^	India	N = 70 (37/33)	1–19	Greulich-Pyle*	− 0.56 (1.33)	− 0.75 (1.53)	− 0.36 (1.04)	Inappropriate estimation method	Inappropriate estimation method
Ye et al. (2014)^46^	China	N = 941 (410/531)	7–14	Demirjian		1.68 (1.29)	1.28 (1.17)	Cohen’s k = 0.89	Cohen’s k = 0.89
				Willems		0.36 (1.19)	− 0.02 (1.18)		
Yusof et al. (2014)^57^	Malaysia	N = 1403 (691/712)	4–24	Willems	0.45 (1.39)	0.58 (1.33)	0.32 (1.43)	Cohen’s k = 0.73	Cohen’s k = 0.98
Zhai et al. (2016)^47^	China	N = 1004 (392/612)	11–18	Demirjian	− 0.57 (1.25)	− 0.47 (1.21)	− 0.63 (1.27)	Inappropriate estimation method	Inappropriate estimation method
				Willems	− 0.83 (1.28)	− 0.54 (1.37)	− 1.01 (1.19)		

*Wrist and hand X-ray; §The ICC variance was estimated using the formula reported in Noble et al.^65^.

Nine different age estimation methods were used, with a clear predominance of the Demirjian approach or its modification (16 out of 23^42,45–47,50,53–56,58–64^) and Willems (13 out of 23^43,44,46,47,49,50,53,55–58,60,62^). Other methods were used less or only once (Cameriere, 5 out of 23^43,48,49,53,58^; Haavikko, 4 out of 23^43,50,58,64^; Smith, 1 out of 23^53^; Nolla 7 out 23^50,51,58,60,61,63,64^; Chaillet, 1 out of 23^55^; Blenkin and Evans, 1 out of 23^62^; Greulich and Pyle, 2 out of 23^52,54^).

Sixteen studies provided complete data for both mean errors and examiner agreements, while eight studies report mean errors in age estimation without complete or usable data regarding the intra-or inter-observer agreement. The precision of the estimation methods was highly variable, with a mean error ranging from a maximum precision of − 0.02 years using the Cameriere method applied to males^43^ to a minimum of − 2.96 years using the Haavikko method applied to females^50^. The inter-examiner agreement ranged between 0.73 and 1 for Cohen’s k/Fleiss’ k and between 0.84 and 1 for ICC; similarly, the intra-examiner agreement ranged between 0.82 and 0.99 for Cohen’s k and between 0.80 and 1 for ICC.

### Study quality assessment (qualitative synthesis)

**Figure 2:**
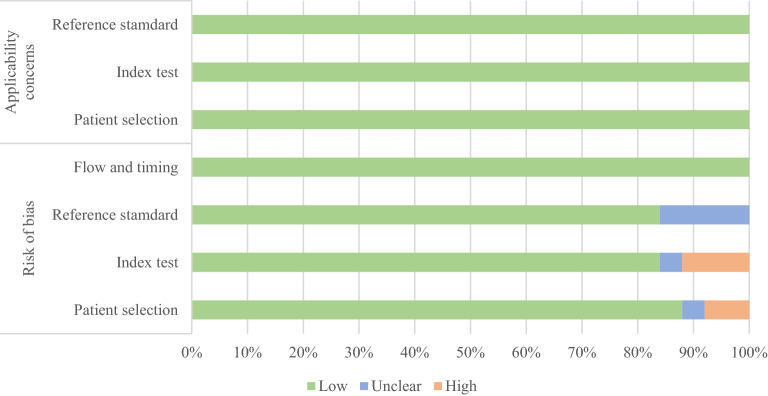
Quality assessment obtained using the QUADAS-2 instrument for the 23 selected studies.

The risk of bias assessment for the selected studies is presented in Table 2 and illustrated in Fig. 2. All studies accurately described the patient selection procedure except for El Bakary et al.^49^ and Javadinejad et al.^53^, in which the procedure was not clearly explained, and Franco et al.^44^, in which the criteria were not reported, so these studies were classified as “unclear”. With respect to the index text, we considered any study that clearly expressed the method of analysis of the radiographs or the experience or number of observers making the measurements as “low” risk. Three studies^49,53,59^ did not provide enough information, while another study was not completely specific^63^. Four studies^44,57,59,63^ did not report how the chronological age was assessed (the reference standard in Fig. 2), and this was interpreted as a risk of bias since a person could be confused or lie about his age. All studies provided good information on flow and timing.

**Table 2:**
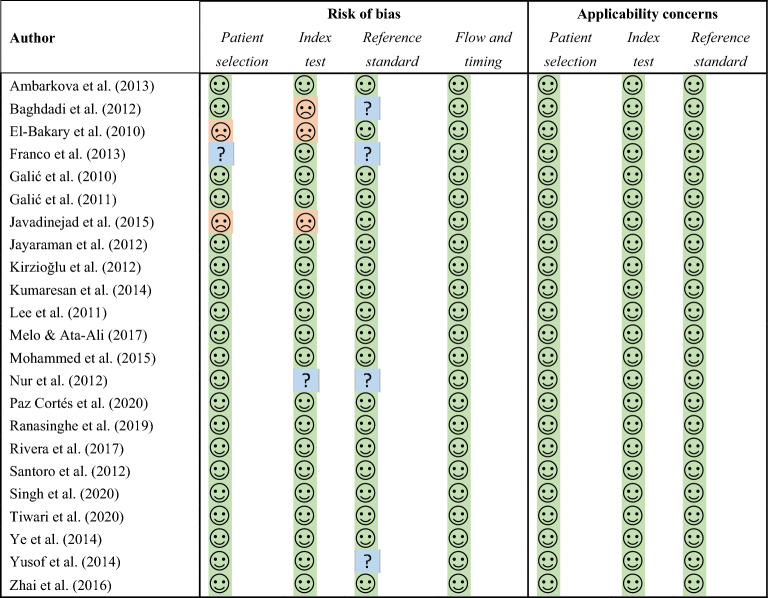
Quality assessment performed using the QUADAS-2 instrument.

Despite the possibility of bias, no study had applicability concerns. All articles met the minimum criterion of regularity in the procedures, as defined by the PICOS/PECOS strategy^66^, and therefore were included in the analysis.

### Meta-analysis of age estimation validity

**Figure 3:**
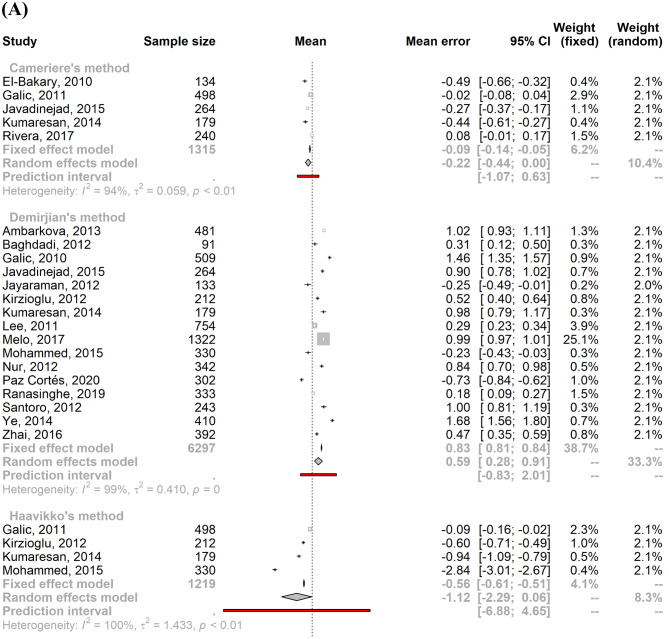

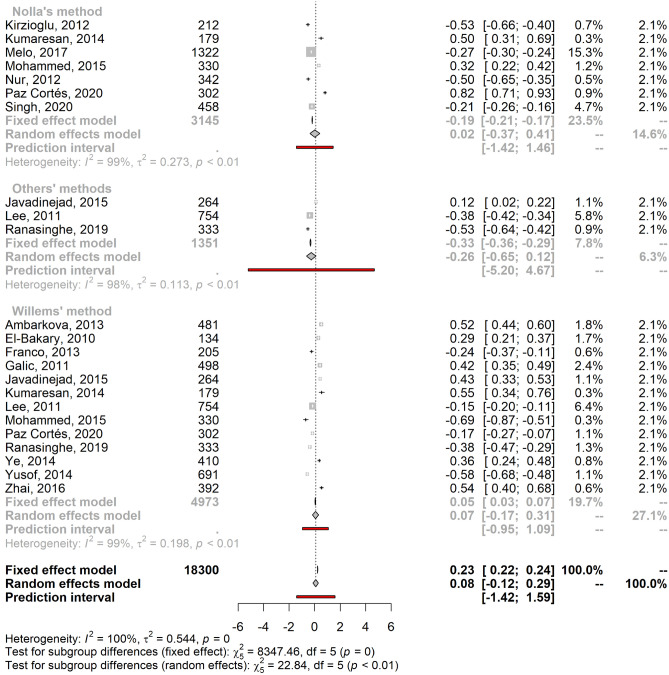

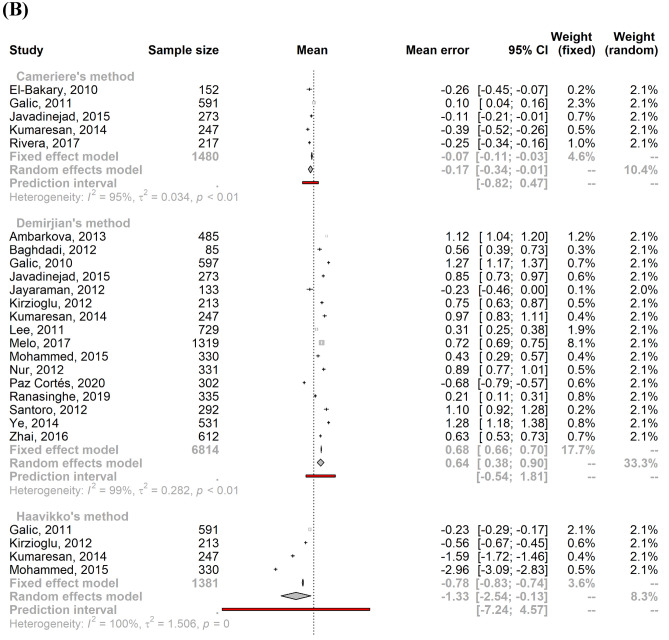

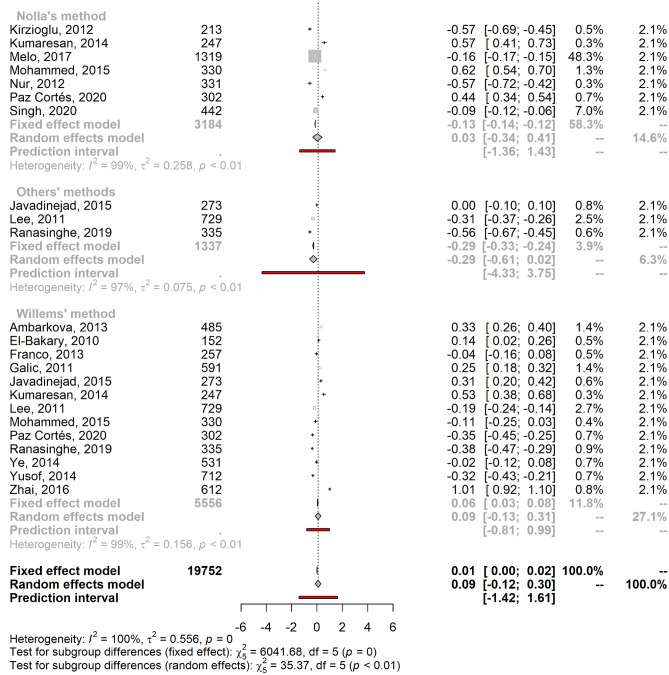
Forest plots showing the pooled mean errors of the age predictions for males (**A**) and females (**B**) by method of age estimation.

Since we found only two studies based on bone maturation indices, we did not produce a meta-estimate of the mean error. Concerning the age estimation validity based on dental maturation indices, significant heterogeneity was found for both males and females (males: I^2^ = 99.6% [95% CI 99.6%; 99.7%]; τ^2^ = 0.54 [95% CI 0.38; 0.86]; females: I^2^ = 99.6% [95% CI 99.5%; 99.6%]; τ^2^ = 0.56 [95% CI 0.38; 0.88]) due to the large sample size and the precision of the included studies. As a result, a mixed-effects model was applied to calculate the pooled mean error of age estimation by sex. The pooled male mean error of the age prediction was 0.08 years (95% CI − 0.12; 0.29), and the pooled female mean error was 0.09 years (95% CI − 0.12; 0.30). Figure 3 shows the stratification by age estimation methods, which are also summarized in Supplementary Methods 1.

Studies that implemented Nolla’s method had a mean error closest to zero with a slight overestimation: mean male age prediction error of 0.02 (95% CI − 0.37; 0.41) and mean female age prediction error of 0.03 (95% CI − 0.34; 0.41). Haavikko’s method was a less accurate method, with a mean error of − 1.12 (95% CI − 2.29; 0.06) and − 1.33 (95% CI − 2.54; − 0.13) for males and females, respectively. Cameriere’s method also underestimated the chronological age and was the only method with a higher absolute mean error for males than females (males: − 0.22 [95% CI − 0.44; 0.00]); females: − 0.17 [95% CI − 0.34; − 0.01]). Generally, Demirjian’s and Willems’s methods tended to overestimate chronological age in both males (Demirjian: 0.59 [95% CI 0.28; 0.91]; Willems: 0.07 [95% CI − 0.17; 0.31]) and females (Demirjian: 0.64 [95% CI 0.38; 0.90]; Willems: 0.09 [95% CI − 0.13; 0.31]).

We included three studies in the “others” category^53,55,62^ for age estimation based on dental maturity (Blenkin & Evans, Chaillet and Smith). These methods underestimated chronological age for both sexes (males: mean = − 0.26; 95% CI [− 0.65; 0.12], females: mean = − 0.29; 95% CI [− 0.61; 0.02]).

For both males and females, the PI overlapped zero for all methods, rendering the difference between estimated and chronological ages not statistically significant. For both genders, Cameriere’s method showed the smallest PI, while the Haavikko and other methods had the widest intervals.

### Meta-analysis of intra- and inter-examiner agreement

**Figure 4:**
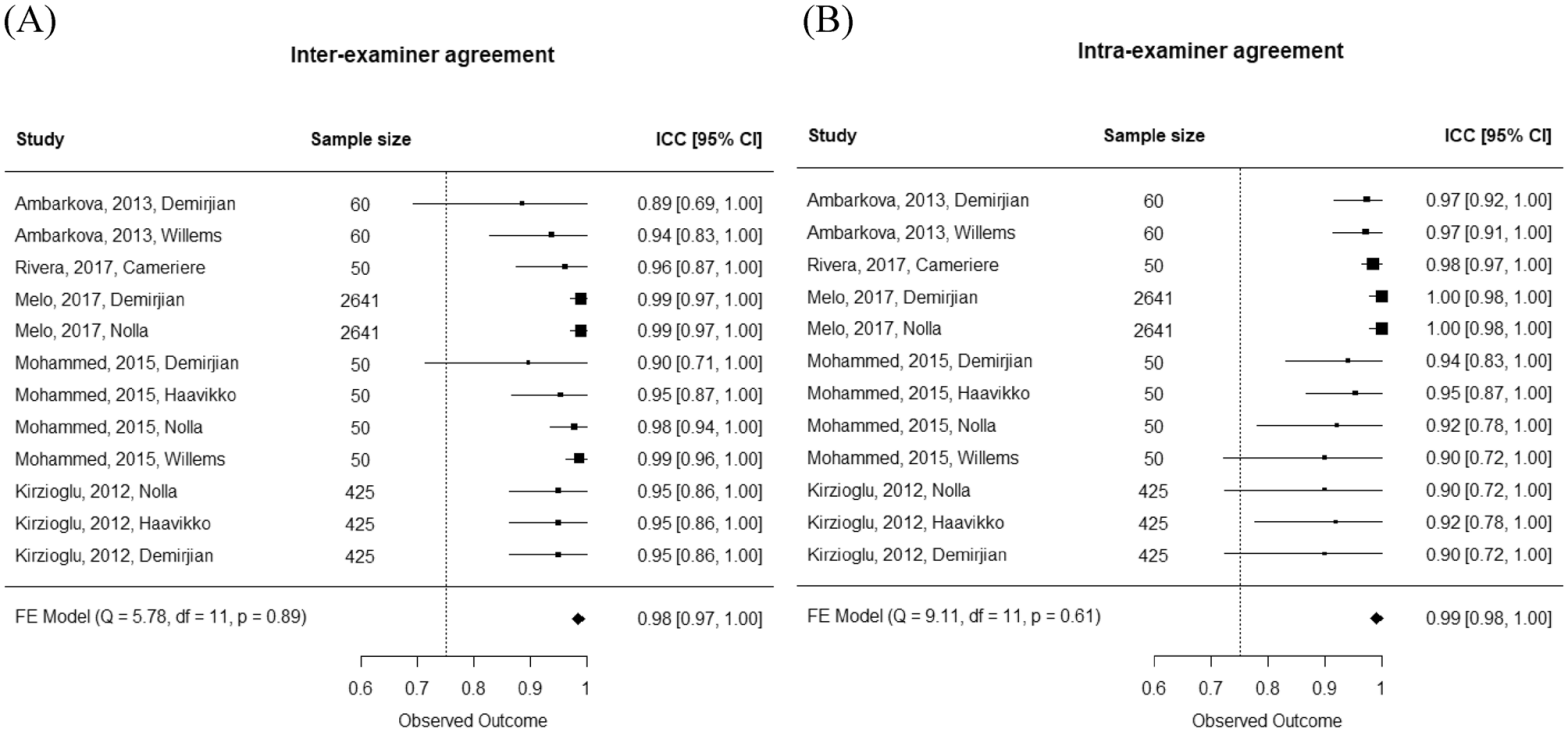
Forest plots showing the pooled inter-examiner (**A**) and intra-examiner (**B**) agreement.

It was not possible to obtain a pooled Cohen’s k (or Fleiss’ k) due to a lack of information on the standard error or variance in the examined studies. Therefore, we compared only studies with ICCs and the studies reporting only the global reliability without stratification by gender. The meta-analytic pooled estimates of inter-examiner and intra-examiner agreement are summarized in Fig. 4.

No heterogeneity was observed in inter-examiner (heterogeneity: Q = 5.78, *p* = 0.888) and intra-examiner (heterogeneity: Q = 9.11, *p* = 0.611) agreement, so a fixed-effects model was used. For inter-examiner agreement, the ICCs ranged from 0.89 to 0.99, and the meta-analytic pooled ICC was 0.98 (95% CI 0.97; 1.00), which was close to perfect reliability. Concerning intra-examiner agreement, the ICCs ranged from 0.90 to 1.00, and the meta-analytic pooled ICC was 0.99 (95% CI 0.98; 1.00), which was also close to perfect reliability.

## Discussion

Age estimation represents one of the most important aspects of dental/skeletal analysis and forensic anthropology, playing a key role in human identification, both in living subjects and to establish identity in human remains^1,2,29^. This meta-analysis provides a comprehensive overview of the current literature on the validity of age estimation methods and reproducibility of maturity indices, in particular those based on dental maturation. Although bone age has been widely used, we found only 2 validation studies on methods based on bone maturity indices that met our inclusion criteria. This low frequency could be due to the evidence that bone maturity indices suffer more from environmental factors than dental ones^23^ and therefore it could be proper to validate each index only in the population in which it is built. The 21 studies on dental maturity indices identified were conducted in different countries with the aim of validating specific methods of age estimation in specific populations. Although the age estimation methods were applied to different populations, the meta-analysis results, stratified by gender and methods, showed similar accuracy. In fact, for both males and females, the prediction intervals obtained for each method spanned zero, indicating that, despite the different prediction intervals and different target populations, all methods can be considered accurate. Significant heterogeneity between studies was observed for both genders as a consequence of the large sample size of the studies and hence of the high level of precision of error estimates. Using a meta-regression model, we investigated whether this heterogeneity might be further explained by differences in characteristics of the studies or study populations such as type of method, publication year, ethnicity, mean age of the study sample, and impact factor of the journal; the I^2^ index still remain very high (99.2%) for both genders (data not shown). The strategies adopted to take into account the heterogeneity between the studies are the estimation of random-effect models and the estimation of prediction intervals to detect a range in which the validity of further studies is expected to be included based on current evidence^67^.

The studies that validated Nolla’s method had a mean age estimation error closest to zero for both males (0.02 years) and females (0.04 years), while Cameriere’s method had the narrowest prediction interval (male PI [− 1.07; 0.63]; female PI [− 0.82; 0.47]). Of the selected studies, Demirjian’s method and its revisited version by Willems were the most frequently used methods for age estimation due to their ease of use, high reproducibility, and accuracy. Both methods tended to overestimate chronological age in males and females, but Willems’ method had a narrow prediction interval, between − 0.95 and 1.09 in males and − 0.81 and 0.99 in females, compared with Demirjian (male prediction interval [− 0.83; 2.01]; female prediction interval [− 0.54; 1.81]).

The Haavikko method had the highest variability, with a prediction interval ranging from − 6.88 to 4.65 for males and from − 7.24 to 4.57 for females. This might be due to the variability in dental maturation among subjects of different ethnic origin^1^, since Haavikko’s method is calibrated on Finnish children, whose dental maturation seems to occur earlier^68^. Recently, Butti et al.^69^ and Mohammed et al.^50^ reached the same conclusion that Haavikko’s method is unsuitable for both Italian and Indian children.

With respect to method reliability, our results showed pooled estimates of reproducibility values close to perfect reliability (about unity), indicating that the methods are highly repeatable by expert examiners. This high reproducibility might be due to positive publication bias, as studies reporting good reliability are more frequently available in the literature than studies reporting poor or no reliability^70^.

The strengths of our research are the adequate number of studies included, the precision of pooled mean errors, and the comprehensive evaluation of all methods and indices based on dental maturity for which, respectively, the validity and reproducibility measures were available in literature. To our best knowledge, this is the first meta-analysis that simultaneously evaluated the validity of dental age estimation methods and reproducibility of maturity dental indices, thereby allowing more informed and safer choices in all medical and legal fields requiring these methods. Finally, the quality assessment of the selected studies was very high: only 10% of studies had an unclear risk or high risk of bias without any concerns about applicability.

However, our evaluation also has some limitations and it shows a partial picture of validity and reproducibility of age estimation methods, due to the strict exclusion criteria applied in order to provide unbiased meta-estimates. We excluded articles without information on both validity and reproducibility outcomes, articles not written in English or Italian, and those where it was impossible to obtain pooled reproducibility estimates of Cohen’s kappa or the ICC due to a lack of information on the variability measure. In addition, some studies used inappropriate methods to estimate reproducibility, as discussed in Ferrante et al.^71^. Lastly, after reading the full texts, we excluded several studies (31 out of 75) with the word “validation” in the title or abstract but that used an inadequate approach to validate the method or no validation at all.

In conclusion, since only two studies based on bone maturity indices reported the validation and reproducibility analysis, it was not possible to perform a meta-analysis for them. All studies reporting methods based on dental maturity indices, which underwent a validation process, were considered in this review and for each method the difference between estimated and chronological age was not significantly different from zero years, highlighting a high validity. Nevertheless, there was a high degree of variability in the precision of the prediction intervals (research focus 1; Supplementary material “Methods”). Furthermore, a high intra- and inter-observer reproducibility of dental maturity indices was observed (research focus 2; Supplementary material “Methods”). The Nolla and Cameriere methods might be recommended as preferred approaches, although the Cameriere method was validated on a smaller sample size than Nolla’s and it requires further testing on additional populations to better assess the mean error estimates by sex. In the development of new methods of age estimation, it will be important to apply rigorous validation and publish a minimum dataset that ensures comparability of validity and reliability between different studies.

## Supplementary Information

Supplementary Information.

## Data Availability

All data is available from the included articles and in the Table 1.
