# Supplementary material for: Validity of age estimation methods and reproducibility of bone/dental maturity indices for chronological age estimation: a systematic review and meta-analysis of validation studies
Source: Sci Rep. 2022 Sep 16;12:15607. doi: 10.1038/s41598-022-19944-5 (PMC9481543; doi:10.1038/s41598-022-19944-5)
Supplement: Supplementary file 1 — Supplementary Information. [file 41598_2022_19944_MOESM1_ESM.docx]

**Validity of age estimation methods and reproducibility of bone/dental maturity indices for chronological age estimation: a systematic review and meta-analysis** **of validation studies**

Marconi V^1^**^#^**, Iommi M^2^**^#^**, Monachesi C^3^, Faragalli A^2^, Skrami E^2*^, Gesuita R^2^, Ferrante L^2^, Carle F^2,4^

*^1^ Postgraduate School of Medical Statistics and Biometry, Department of Biomedical Sciences and Public Health, Università Politecnica delle Marche, 60126 Ancona, Italy*

*^2^ Center of Epidemiology, Biostatistics and Medical Information Technology, Department of Biomedical Sciences and Public Health, Università Politecnica delle Marche, 60126 Ancona, Italy*

*^3^ Department of Pediatrics, Università Politecnica delle Marche, 60123, Ancona, Italy*

*^4^ National Centre for Healthcare Research and Pharmacoepidemiology, 20126, Milano, Italy*

**^#^ Contributed equally to this paper and should be considered joint first author**

**^*^ Corresponding author:** Edlira Skrami, email: e.skrami@staff.univpm.it

**METHODS**

*Research focus*

This systematic review was conducted to: 1) evaluate the validity of age estimation methods based on bone and dental maturity indices; and 2) evaluate the reproducibility of bone and dental maturity indices intra- and inter-observers. The methodology was developed in accordance with the Preferred Reporting Items for Systematic Reviews and Meta-Analysis (PRISMA) statement. This review was registered in the International Prospective Register of Systematic Reviews (PROSPERO) database, accession number CRD42021276534.

*Literature search strategy*

Literature searching was performed independently by two researchers (VM and CM) between 1^st^ and 15^th^ February 2022 using the PubMed and Google Scholar databases. The two authors discussed the search criteria and selected studies reporting the validity of dental and bone age estimation methods and reproducibility of the maturity indices published up to 31^st^ December 2021.

The search strategy included appropriate keywords and followed the syntax rules for each database. Free terms related to age estimation, dental or bone methods, validity, and reproducibility were used to search the titles and abstracts combined with Boolean operators (OR, AND) (see **Supplementary Methods 2**). There were no date restrictions, and we considered only studies involving humans and written in English or Italian. Moreover, we considered only the studies reporting both the validation process and the reproducibility assessment, to limit the potential confounding bias caused by evaluating the validity and reproducibility of the same index on two different samples from different populations. Articles appearing in more than one database search were considered once.

*Eligibility criteria*

Eligible studies had to fulfil PICOS/PECOS (Participants, Intervention/Exposure, Comparison, Outcome, Study design) criteria as follows: P: all human subjects submitted to age estimation; I: dental and/or skeletal radiography as a diagnostic tool; C: (1) comparison between chronological (date of radiography – date of birth) and estimated age; (2) comparison inter-observers and intra-observer in radiography evaluation; O: (1) mean error of age estimation methods (estimated age - chronological age); (2) Cohen’s K for qualitative methods, Concordance correlation coefficient (CCC) or Intra-class correlation coefficients (ICC) for quantitative methods; S: cross-sectional human studies. Exclusion criteria were: clinical trials, case reports, letters, commentaries, editorials and reviews; studies without information on both outcomes 1 and 2; and studies with missing reference population information.

The same two examiners independently initially selected the articles from analysis of the titles and abstracts. Subsequently, full-text versions of papers that appeared to meet the inclusion criteria were retrieved for confirmation of eligibility. The results of both authors were compared, and any disagreement was solved through discussion with a third investigator (LF).

*Data extraction*

An electronic spreadsheet was created to standardize data extraction. This spreadsheet included the: author(s), title, year of publication, journal, index/method of interest, country, sample size, age range, sex, radiological examination performed, error measure results (mean and standard deviation (SD) of difference between estimated age and chronological age), and inter- and intra-examiner agreement coefficients (Cohen’s K, ICC, CCC). The two examiners read the full articles and extracted data independently. Discrepancies in data extraction were solved through discussion with LF.

*Study quality assessment (qualitative synthesis)*

The methodological quality of the selected studies was evaluated separately and independently by the two examiners (VM, CM) using the QUADAS-2 tool^66^. Disagreements between reviewers were solved by discussion with a third reviewer (LF).

QUADAS-2 contains four elements that define patient selection, the index test, the reference standard, and flow and timing. Each question can be answered “Yes”, “No”, or “Unclear”. After evaluation, the two examiners compared their analysis and classified the studies as low, high, or unclear risk according to QUADAS-2 criteria.

*Statistical analysis*

Studies’ results were summarized using the pooled estimates of the mean age estimation error; separate analyses were performed for males and females, stratifying by age estimation methods. Methods were grouped into an “others” category if they were found in less than 3 studies. Pooled estimates of intra- and inter-examiner agreement were also calculated. When the ICC standard error was not reported, we estimated the ICC variance using the formula reported in Noble et al.^65^.

Statistical heterogeneity was analyzed to determine the feasibility of summing the results of different studies considered eligible for meta-analysis. The heterogeneity variance τ^2^, which quantifies the variance of the true effect sizes, was estimated along with the prediction interval (PI)^67^. We assessed heterogeneity by calculating the I² statistic, which represents the percentage of variance in the estimated effects due to heterogeneity rather than chance. An I² statistic >50% was considered significantly heterogeneous. When there were <5 studies or studies were substantially heterogeneous, we used a random-effects model in accordance with the Cochrane Handbook for Systematic Reviews of Interventions^70^. We used forest plots to show the estimated effects with 95% confidence intervals (CIs) for individual studies and pooled results. All statistical analyses were performed in R version 4.1.0 using the *meta* and *metafor* packages.

**Supplementary Information**

**Supplementary Methods 1 – Description of age estimation methods considered in the meta-analysis.**

1. DEMIRIJIAN’S AGE ESTIMATION METHOD^24^: The method developed by Demirjian involves the study, by OPT, of seven elements of the left mandibular hemiarch: I1 (central incisor), I2 (lateral incisor), C (canine), PM1 (1st premolar), PM2 (2nd premolar), M1 (1st molar), and M2 (2nd molar), excluding the third molar as it is considered unreliable because it is subject to anomalies in shape, eruption, and formation compared with the other teeth. This method requires, unlike other methods, the prior determination of the sex of the subject examined, which implies knowledge of or the need to ascertain this information. Subsequently, for each tooth examined, the evolutionary stage reached is identified with comparative reference to one of the eight stages of maturation provided by the appearance of calcification points (stage A) until the closure of the root apices (stage H). Each maturational stage of each tooth is assigned a score differentiated by sex. The sum of the scores related to each tooth creates a score expressive of the global dental maturity, which when reported on special conversion tables provides the dental age.
2. NOLLA’S AGE ESTIMATION METHOD^72^: Nolla devised a method of age estimation by evaluating the calcification of the permanent dentition. The calcification of permanent dentition is divided into stages: 1) absence of crypt; 2) presence of crypt; 3) initial calcification; 4) 1/3^rd^ crown completed; 5) 2/3^rd^ crown completed; 6) crown almost completed; 7) crown completed; 8) 1/3^rd^ root completed; 9) 2/3rds of root completed; 10) root almost completed–open apex; 11) apical end of root completed for each group of teeth like incisors, canine, premolars, and molars of the maxillary and mandibular arches separately. The radiograph of the individual is matched to a comparative figure given by Nolla. Each tooth is recorded with a reading and a sum made for maxillary and mandibular teeth; later, the total is compared with the table. Separate tables are provided for boys and girls and including or excluding third molars. The opts of younger children and older children are matched with the calcification figures provided by Nolla. Seven mandibular teeth and seven maxillary teeth in the left quadrant are recorded for their stage of calcification with a reading. The scores for the seven mandibular and seven maxillary teeth are totalled to derive a score for fourteen maxillary and mandibular teeth. Later, the sum is matched with the table given by Nolla for boys and girls separately.
3. HAAVIKKO’S AGE ESTIMATION METHOD^73^: Haavikko utilized a modified version of the dental developmental stages of Gleiser and Hunt with the number of stages reduced from 15 to 12 (six each for crown and root formation) to study the ages of tooth formation in Finnish children. From data derived by evaluating all the maxillary and mandibular teeth, Haavikko constructed gender-specific tables of age medians and dispersions for each stage of tooth development. Age medians for each tooth assessed are summed and divided by the number of teeth assessed to directly give the dental age.
4. GREULICH AND PYLE’S AGE ESTIMATION METHOD^74^: The method originally proposed and developed by Todd in 1937, and subsequently extended by his pupils Greulich and Pyle in 1959, is a qualitative method for determining the maturity of children and adolescents. Using X-rays of the left hand and wrist in a flat position and with a posterior view, the bone maturity is determined from the degree of mineralization of the bones in the wrist (carpus), the development of the metacarpal bones and phalanges, and the degree of fusion of the distal epiphyses in the ulna and radius. In practical terms, the bones that appear in the X-ray are read in order to determine the presence or absence of certain carpals, to assess the degree of ossification of the epiphysis, to establish the shape and size of the bones, and to estimate the degree of fusion of the epiphyses and their respective diaphysis. Considering this information, and to avoid confusion when irregularities in the order of appearance of the bones occur, age is determined based on greater similarity with the standard in the atlas. This atlas consists of separate reference images for boys and girls aged 0-18 (boys) or 0-19 years (girls) at various intervals (3 months-1 year). Images are accompanied by an explanation of the gradual age-related changes in the bones at a given age and separate bone ages calculated for each bone. Due to the natural variability of the bone age of different bones in one individual, in some bones, it is often more or less advanced than the standard it is intended to represent. Bone age is calculated by comparing the non-dominant wrist radiographs of the subject with the nearest matching reference radiographs provided in the atlas. GP is the most popular method used by clinicians and radiologists, as the assessment by GP is relatively quick and easy to learn.
5. SMITH’S AGE ESTIMATION METHOD^75^: In 1991, Smith modified the method by Moorrees, Fanning, and Hunt, which describes each tooth individually and averages all the ages in such a manner that the median corresponds to the dental age. Note that in this method, apical closure does not have a value assigned as a marker of dental age. What is interesting about this method is that it focuses specifically on age prediction. This numerical method has elevated and very significant approximation values; it is also easy to use, given that its main objective is age prediction.
6. WILLEMS AGE ESTIMATION METHOD^76^: Willems et al. revised Demirjian’s method by creating new scoring tables based on their patient data. Demirjian’s maturity scores were adapted using weighted ANOVA on the data of the Belgian Caucasian sample. The conversion of the maturity score to dental age was removed, making this method practical to use while retaining the advantages of Demirjian's original technique. Willems method considers the developmental stages of the seven left permanent mandibular teeth. A score is obtained for each tooth from sex-specific tables. The seven teeth are labeled A to H on the left quadrant of the mandible in the panoramic view with the assistance of the reference illustrations and radiographs. Finally, Willems dental age estimation is obtained using the score corresponding to each letter code for boys and girls. This resulted in new tables for boys and girls with age scores directly expressed in years. Calculating the overall maturity score by summing the adapted scores for the seven mandibular teeth directly results in the estimated dental age.

**Supplementary Methods 2 - Electronic database used and search strategy**

**PubMed**

- VM: *(“Age estimat*”[Title/Abstract] OR “age determination”[Title/Abstract]) AND (dental OR skeleton) AND (accura* OR validit*) AND reproducibility [Title/Abstract]*

*Filters: Full text*

- - 21 articles
- CM: *age[TI] AND estimation[TI] AND (dental OR bone) AND method AND validity AND reproducibility*

*Filters: Full text*

- - 30 articles

**Google Scholar**

- VM: ("Age estimation" [TI] OR "age determination" [TI]) AND (dental[TI] OR skeleton[TI]) AND (accuracy[TI] OR validation[TI]) AND (reproducibility[TI] OR reliability[TI] OR “agreement”[TI]) AND ( "opt" OR "ray" OR "tomography") AND "HUMAN"
  - *145 articles*
- CM: *“AGE ESTIMATION” [TI] AND (DENTAL[TI] OR BONE[TI]) AND ("AGREEMENT"[TI] OR "RELIABILITY"[TI] OR “REPRODUCIBILITY”[TI]) AND "HUMAN" [TI] AND (VALID*[TI] OR "ACCURACY"[TI])*
  - *237 articles*
